# Membrane Models and Experiments Suitable for Studies of the Cholesterol Bilayer Domains

**DOI:** 10.3390/membranes13030320

**Published:** 2023-03-10

**Authors:** Ivan Mardešić, Zvonimir Boban, Witold Karol Subczynski, Marija Raguz

**Affiliations:** 1 Department of Medical Physics and Biophysics, University of Split School of Medicine, 21000 Split, Croatia; imardesi@mefst.hr (I.M.); zvonimir.boban@mefst.hr (Z.B.); 2 Faculty of Science, University of Split, Doctoral Study of Biophysics, 21000 Split, Croatia; 3 Department of Biophysics, Medical College of Wisconsin, Milwaukee, WI 53226, USA; subczyn@mcw.edu

**Keywords:** plasma membrane, cholesterol, phospholipids, liposomes, supported lipid bilayer, cholesterol bilayer domains, cholesterol crystals, saturation recovery electron paramagnetic resonance, atomic force microscopy, X-ray diffraction

## Abstract

Cholesterol (Chol) is an essential component of animal cell membranes and is most abundant in plasma membranes (PMs) where its concentration typically ranges from 10 to 30 mol%. However, in red blood cells and Schwann cells, PMs Chol content is as high as 50 mol%, and in the PMs of the eye lens fiber cells, it can reach up to 66 mol%. Being amphiphilic, Chol molecules are easily incorporated into the lipid bilayer where they affect the membrane lateral organization and transmembrane physical properties. In the aqueous phase, Chol cannot form free bilayers by itself. However, pure Chol bilayer domains (CBDs) can form in lipid bilayer membranes with the Chol content exceeding 50 mol%. The range of Chol concentrations surpassing 50 mol% is less frequent in biological membranes and is consequently less investigated. Nevertheless, it is significant for the normal functioning of the eye lens and understanding how Chol plaques form in atherosclerosis. The most commonly used membrane models are unilamellar and multilamellar vesicles (MLVs) and supported lipid bilayers (SLBs). CBDs have been observed directly using confocal microscopy, X-ray reflectometry and saturation recovery electron paramagnetic resonance (SR EPR). Indirect evidence of CBDs has also been reported by using atomic force microscopy (AFM) and fluorescence recovery after photobleaching (FRAP) experiments. The overall goal of this review is to demonstrate the advantages and limitations of the various membrane models and experimental techniques suitable for the detection and investigation of the lateral organization, function and physical properties of CBDs.

## 1. Introduction

**Figure 1:**
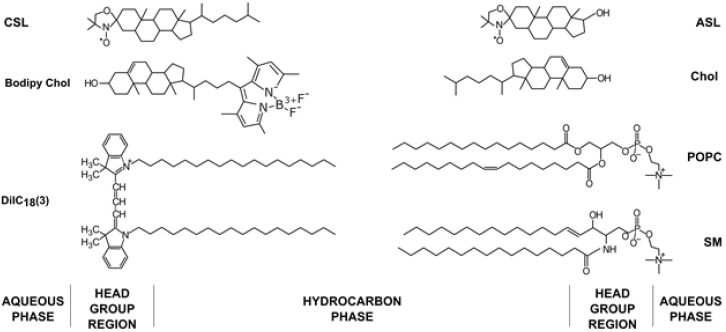
Chemical structures of lipids (1-palmitoyl-2-oleoyl-glycero-3-phosphocholine (POPC), SM and Chol), fluorescent dyes (Bodipy Chol and 1,1′-dioctadecyl-3,3,3′,3′-tetramethylindocarbocyanine (DiIC_18_(3))) and spin labels (androstane spin label (ASL) and cholestane spin label (CSL)) used in the membrane model experiments with their approximate locations in the membrane bilayer.

A plasma membrane (PM) is a complex structure that separates the cell from its environment. It contains different lipids, mostly phospholipids (PLs), sphingolipids and cholesterol (Chol), but also membrane proteins and carbohydrates [1,2]. PLs differ with respect to the headgroup, hydrocarbon chain length and degree of unsaturation. The properties of PMs change depending on the type of PLs they contain and the differences in the Chol content [3]. Chol plays many roles in PMs, influencing the membrane thickness and rigidity [4,5], domain formation [6,7] and cell signaling [8]. Chol is an amphiphilic molecule consisting of a large, nonpolar rigid planar fused ring structure with a smooth and a rough side, a short flexible isooctyl tail and a small hydroxyl (−H) hydrophilic moiety [4] (Figure 1). With such a structure, the Chol molecule naturally orients itself vertically in the PL bilayer, with its head among the polar heads of other PLs and the fused ring structure tugged inside the acyl chain region of the lipids. This enables the Chol molecule to regulate the lateral organization of PLs and influence the membrane physical properties at different membrane depths. These regulatory functions include altering the bilayer transition temperature [9] and the formation of certain membrane phases and domains [10,11,12]. In the last few decades, raft domains (domains that are rich in Chol and sphingolipids and span both bilayer leaflets) attracted researchers’ attention. Raft domains control many complex functions of PMs, such as signal transduction, protein sorting and lipid trafficking [13,14,15,16,17]. This research is well documented and reviewed [17]. PMs are a more complex system compared to model membranes due to the heterogeneous distribution of proteins and the interactions of lipid components with proteins and the cytoskeleton [18]. The properties of Chol differ in PMs compared to model membranes. Recent studies indicate that Chol moves 1.2 times faster in model membranes compared to the PLs and sphingomyelin (SM) and 2 times faster in live cells compared to the PLs and SM [19]. The same study showed two-component diffusion in PMs, the fast one and one similar to PLs. When the Chol analog was localized in the outer leaflet, the fast diffusion disappeared. This suggests asymmetric Chol diffusion and localization in PMs.

**Figure 2:**
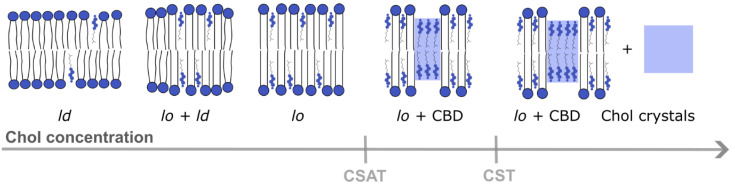
Different membrane domains (*l*_d_—liquid-disordered domain, *l*_o_—liquid-ordered domain) formed in a lipid bilayer at different Chol concentrations from lower to higher content. CBDs start to form above the CSAT, and Chol precipitates in the form of Chol crystals above the CST level [21].

When the Chol content in the lipid bilayer exceeds a Chol saturation threshold (CSAT) (around 50 mol% Chol), pure Chol bilayer domains (CBDs) are formed within the bulk bilayer. CBDs span both membrane leaflets [20] and are stabilized by the surrounding PL bilayer saturated with Chol [21] (Figure 2). Contrary to lipid rafts, these domains and their functions are less investigated and documented. The eye lens fiber cells contain large amounts of Chol, and it was shown that CBDs are present in the lens lipid membranes [22], forming the buffering capacity, which ensures that these membranes are always saturated with Chol. Because of that, it is argued that CBDs guarantee the homeostasis of the fiber cell PMs, fiber cells themselves and the entire eye lens [23,24,25,26].

A further increase in the Chol content in the lipid bilayer (above the Chol solubility threshold (CST), ~66 mol%, (Figure 2) induces the formation of Chol crystals which can activate an inflammatory cascade, leading to the development of atherosclerosis. The CST is a total amount of Chol that can be accommodated by the lipid bilayer membrane, both in the PL bilayer saturated with Chol and in CBDs. Thus, the appearance of CBDs in biological membranes other than the eye lens fiber cell membranes is usually treated as a sign of pathology [27]. 

In this review, we will present different techniques which allow the discrimination of CBDs in model and intact membranes. We will focus on membrane models which can provide information on the major CBD properties. Finally, we will discuss the future direction of CBD research.

## 2. Liposomes with High Chol Content

**Figure 3:**
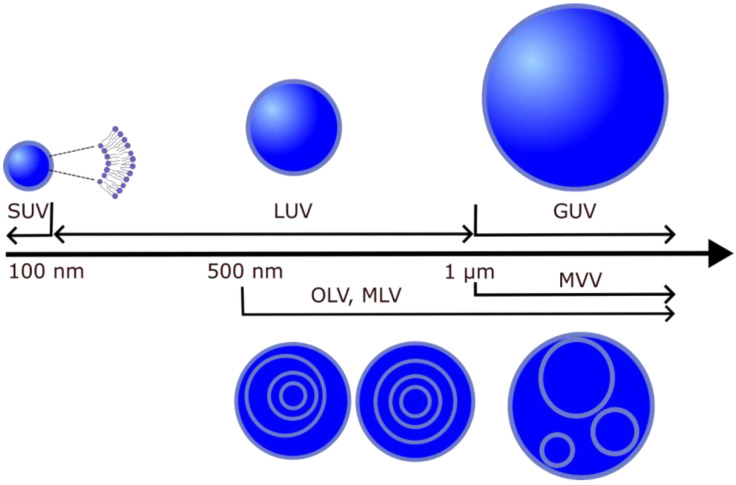
Different types of lipid vesicles depending on their size and structure.

Liposomes (lipid vesicles) are structures with at least one lipid bilayer enclosing an aqueous solution. Because they enable research under controlled conditions, they are often used as biological membrane models for investigating the physical properties of membranes [28,29], mimicking cell interactions [30,31,32], drug delivery [33], interactions with toxins [34] and nanoparticles [35]. In addition, a possible application of liposomes is to use them to build cell-like systems that form whole-cellular models [36]. Depending on their lamellarity, lipid vesicles can be unilamellar (single bilayer), multilamellar (MLVs, more than one concentric bilayer), oligolamellar (OLVs, smaller vesicles within a larger one) or multivesicular vesicles (MVVs, bilayers within other bilayers that are not concentric). Unilamellar vesicles are further classified according to their size into small (SUV) (<100 nm), large (LUV) (>100 nm and <1 μm) and giant (GUV) (>1 μm) unilamellar vesicles. Depending on the target vesicle type, different methods are used to produce them (electroformation, hydration, sonication, extrusion, etc.) (Figure 3) [37,38]. In the following sections, we will first discuss the protocols for the preparation of lipid vesicles with Chol content above the CSAT and, later, the experimental techniques used to study these models. 

### 2.1. Liposome Preparation

#### 2.1.1. MLVs, SUVs and LUVs

In order to form MLVs, lipids are usually first dissolved in an organic solvent (chloroform or chloroform/methanol). The lipid mixture is deposited in the test tube or on the substrate and dried using inert gas, rotary evaporation or lyophilization until the lipid film is formed. A vacuum is used to remove the residual organic solvent. The lipid film is then hydrated with an aqueous solution at a temperature above the highest transition temperature of lipids in the mixture. During hydration, the lipid sheets start swelling and detach to form MLVs. Shaking, vortexing or stirring is needed to enhance the liposome formation [37]. The problem with this approach is the appearance of artificial Chol crystals during the drying process, especially for a Chol concentration above the CSAT. Consequently, the amount of Chol incorporated into the vesicle bilayer is lower than intended because a portion of Chol from the mixture forms Chol crystals which do not participate in the following vesicle formation. In order to differentiate between these two values, we introduced the terms mixing and molar Chol ratios. The mixing ratio defines the ratio of Chol to other lipids dissolved in the organic solution, and the molar ratio is the ratio of the actual amount of Chol to other lipids incorporated into the vesicle bilayer. Using these terms, it follows that Chol demixing causes problems in the sample preparation because the molar ratio in the bilayer is lower than the mixing ratio. 

**Figure 4:**
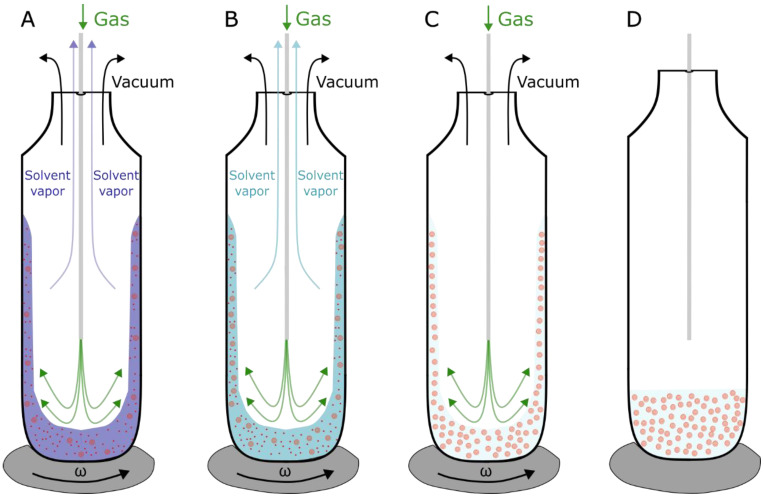
Schematic depiction of the RSE method. (**A**) Organic solvent (chloroform) (blue) containing lipids (red) is mixed with aqueous solution, and removal of organic solvent is achieved by vortexing the solution under vacuum. The process is made more efficient by adding a flow of inert gas (argon) (green) pushing the organic solvent vapors out. (**B**) MLVs start to form. The vacuum pressure is set below the organic solvent evaporation point but higher than the evaporation point of water. (**C**) Exchange is finished when all the organic solution is evaporated and only aqueous solution containing formed MLVs is left. (**D**) When MLVs are formed, vortex is turned off, and vacuum and gas flow pipes are closed. As reported in [40], the whole process takes less than a minute.

In order to avoid the Chol demixing artifact, a method called the rapid solvent exchange (RSE) can be utilized for the production of MLVs (Figure 4) [39,40]. This method bypasses the dry phase by mixing the lipids dissolved in an organic solvent with an aqueous solution. Applying a vacuum in combination with inert gas flow, the organic solvent is gradually evaporated, leaving only MLVs in an aqueous solution. To confirm the advantages of the RSE method over the film deposition method, differential scanning calorimetry experiments were performed on MLV dispersions for various Chol contents, including those above the CST. We showed that using the RSE for the production of MLVs with Chol contents below the CST, no transition peaks were recorded. A single broad transition peak at 86 °C, representing a transition of a monohydrate to an anhydrous crystal, is observed only for mixtures with Chol content above the CST. Conversely, the dispersions obtained using the film deposition method displayed two peaks. Aside from the broad peak at 86 °C, an additional peak appeared at ~36 °C, representing transformations of one form of anhydrous crystal to another. This is attributed to artificial Chol demixing, resulting in Chol crystals that do not participate in a further vesicle formation. Consequently, the experiments confirm the usefulness of the RSE in terms of bypassing the Chol demixing artifact [21].

SUVs and LUVs are used as intermediate steps in protocols for GUVs production [41] or the formation of supported lipid bilayers (SLBs) [42]. In order to form SUVs and LUVs, MLVs are usually produced first. The sonication of the MLV suspension produces SUVs with diameters in the range of 15 to 50 nm. During extrusion, the MLVs suspension is passed through a polycarbonate filter and a membrane with pores of different sizes. The extruder is positioned on a hot plate, with the temperature set above the highest transition temperature of all the lipids in the mixture. To achieve a homogeneous unilamellar vesicles population, the MLV suspension has to be passed through the pores around 15 times. The final vesicle diameters are determined by the size of the membrane pores. It should be noted that formed vesicles usually have a somewhat larger average diameter than the pores, if the pore size is smaller than 200 nm [43]. This is due to the elasticity of the vesicle membranes, allowing them to squeeze through pores narrower than their nominal diameter. The size distribution of produced unilamellar vesicles can be verified using dynamic light scattering. Although less common, the freeze–thaw and the hand-shaking methods can also be used for SUV and LUV formation [37].

#### 2.1.2. GUVs

GUVs are widely used for the investigation of membrane properties due to their size being similar to the sizes of eukaryotic cells. Most often, they are analyzed using optical microscopy techniques. Different approaches have been attempted for GUV production [37,44,45,46,47]. One of the first attempts was reported by Reeves and Dowben in 1969 [29]. The lipid mixture was deposited onto the surface and dried to form a lipid film. The lipid film is then rehydrated, and the solution is stirred to form GUVs. Due to osmotic pressure, the aqueous solution is driven between the lipid bilayer stacks. It is energetically unfavorable for the hydrophobic chains of lipids to be exposed to an aqueous solution, so lipid bilayers spontaneously close into spherical vesicles. Although simple and straightforward, the method produces a low GUVs yield, with lots of defects and a high proportion of MLVs [48]. To address these shortcomings, Angelova and Dimitrov invented the electroformation method which is the most common method used today. In addition to hydration due to osmosis, the method also involves an external electric field applied to the lipid film to promote swelling and vesicle formation [49]. The exact theoretical mechanism of electroformation is not yet completely understood, but it is believed that the electric field affects the lipid swelling through direct electrostatic interactions, the redistribution of counterions, changes in the membrane surface and line tension, and electroosmotic flow effects [50].

**Figure 5:**
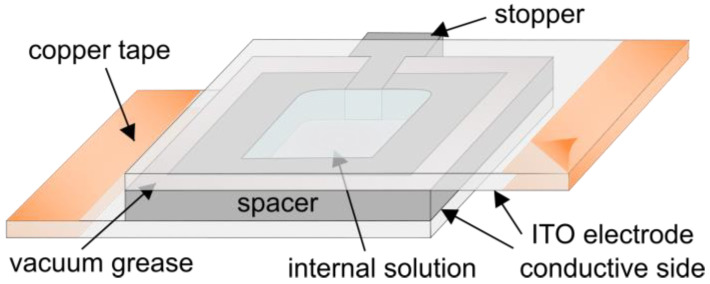
Schematic drawing of a chamber used in electroformation experiments.

The electroformation protocol starts with a mixture of lipids dissolved in an organic solvent. The mixture is spread on conductive electrodes and the bulk of the solvent evaporates shortly after. The remaining traces of the solvent are removed using a vacuum or a flow of inert gas. To form an electroformation chamber (Figure 5), a spacer is attached to the electrodes using vacuum grease. 

After being assembled, the chamber filled with an internal solution is connected to an alternating current waveform generator in order to produce an alternating electric field inside the chamber. Copper tape is often attached to the edges of the electrodes in order to provide better contact with the wires leading to the function generator. The successfulness of the final electroformation depends on lots of factors, such as the choice of electrode material, the film deposition method, the procedure for solvent removal, the lipid composition and internal solution, the choice of electrical parameters, the temperature and the duration of electroformation. The influence of the electroformation parameters on the final result is covered in more detail in a previous review by our group [51].

In order to achieve the best electroformation results, our group conducted experiments in the Chol regime above the CSAT using binary and ternary mixtures of POPC/Chol and POPC/SM/Chol [52,53]. The optimal voltage was found to be in the range of 2 to 6 V and the frequency in the range from 10 to 100 Hz. Lipid thickness is also a parameter that needs to be considered for optimal GUV electroformation. Using spin-coating to achieve reproducible lipid film thicknesses, the highest GUVs successfulness was achieved for thicknesses of approximately 30 nm [53].

Protocols containing steps involving a dry phase result in a lipid film containing Chol crystals formed due to the Chol demixing artifact. Unfortunately, the RSE method cannot be directly used in this case because it produces a solution of multilamellar vesicles. A possible solution was offered by Baykal-Caglar et al. who spread the RSE suspension on an electrode and then placed it in a chamber with a humidity of 55% for 22–25 h to obtain a damp lipid film. Because the lipid film was never completely dry, the Chol demixing artifact was significantly reduced [41].

### 2.2. Experimental Techniques Utilizing Liposome Models with Particular Focus on CBDs

#### 2.2.1. X-ray Diffraction

Barrett et al. used a specific type of MLVs called highly oriented multilamellar membranes to identify pure Chol domains by X-ray diffraction [54]. 1,2-Dimyristoyl-*sn*-glycero-3-phosphocholine/Chol mixtures with a Chol content ranging from 0 to 60 mol% were used. Highly ordered Chol domains were observed when the Chol content exceeded 40 mol%. These domains were not referred to as CBDs in the article but as immiscible cholesterol plaques. One out-of-plane Bragg peak was observed for Chol concentrations up to 40 mol% and an additional one for concentrations above 40 mol%. The second peak indicates the presence of a Chol bilayer coexisting with the lamellar membrane structure. The in-plane signals were used to infer the type of the Chol structure. At 40 mol% Chol, the pattern was fitted to a monoclinic structure. At a Chol content of 60 mol%, a triclinic structure was observed. The height of the pure Chol domain was estimated to be 26.5 to 32.5 Å. The Chol molecule is 17 Å long, so these values are compatible with the Chol bilayer (the CBDs may be tilted). An important aspect to mention is that all measurements were performed in a humidity chamber with 50% relative humidity. When the humidity was increased, the signal corresponding to pure Chol domains disappeared at a Chol concentration of 40 mol%.

#### 2.2.2. SR EPR Spin Labeling

Using EPR techniques, the CBD as a pure Chol domain can be detected only with Chol analogs, such as an ASL and a CSL (see Figure 1 for spin labels structures). However, conventional and saturation recovery EPR spectroscopy alone cannot discriminate CBDs from the surrounding PL bilayer containing Chol because the spectral characteristics of the Chol analogs are very close (undistinguished) in these two environments [55,56]. Fortunately, a paramagnetic relaxation agent, hydrophobic (dissolved in membranes) molecular oxygen and polar water soluble Ni(II) diethylene diamine diacetic acid exist. The effect of these relaxation agents is especially pronounced on the spin lattice relaxation time of the ASL and CSL and is proportional to the bimolecular collision rate between the relaxation agent and the nitroxide moiety of these spin labels. In turn, bimolecular collisions depend on the local relaxation agent concentration and the diffusion coefficient around the nitroxide fragment, and thus the local product of the relaxation agent concentration and diffusion. Because these two components can be very different in the different environments surrounding the nitroxide moieties of the ASL and CSL, the observed EPR saturation signal is giving two separated components (which can be fitted by two exponential functions) [55]. In our discrimination approach, the effect of molecular oxygen on the spin lattice relaxation time of the nitroxide moiety of the ASL (located in the membrane center) is very strong and different in CBDs and the surrounding PL bilayer, allowing to discriminate the CBD. The surface-located nitroxide moiety of the CSL is strongly affected by water-soluble Ni(II) diethylene diamine diacetic acid but differently when CSL is present in CBDs and in the surrounding PL bilayer. These two methodological approaches are described in detail in [55] and were used to discriminate CBDs in model [57], lens lipid [22,23,58] and intact membranes [59].

#### 2.2.3. Fluorescence Microscopy

**Figure 6:**
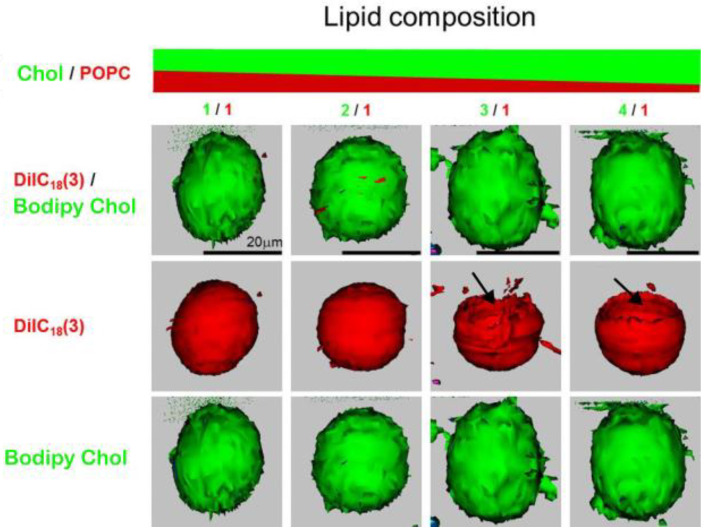
Confocal fluorescence images of GUVs made of Chol/POPC mixtures, with mixing ratios from 1 to 4 and recorded at 22 °C. Each vesicle contains PL and Chol analog probes, DiIC_18_(3) (red) and Bodipy Chol (green), respectively. In the upper row, each image is an overlay of two simultaneously acquired fluorescence signals from DiIC_18_(3) and Bodipy Chol. These same data are presented in the middle and bottom rows, but fluorescence signals originate from DiIC_18_(3) in the middle row and Bodipy Chol in the bottom row, respectively. We collected green and red signals in an independent mode. Each fluorophore was excited by its own individual excitation wavelength, and the resultant emission was collected, first for Bodipy Chol (green) and then for DiIC_18_(3) (red) data. This sequence was repeated over different Z-levels. This enables each fluorophore to be captured independently and in microseconds apart from each other. To gain a three-dimensional distribution of DiIC_18_(3) and Bodipy Chol in the GUV, Z-scan images were converted into surface-rendered images. Three-dimensional renderings of the Z-stack images were performed under the same settings, including threshold (lower limit set at 0.05 for both green and red) orientation, projected image brightness, etc. Arrows show the lack of fluorophore in certain parts of the vesicles. Data for Figure 6 are reproduced with permission from [20]. Copyright 2023, Springer nature BV.

The Chol demixing artifact was encountered by our group as well while using confocal microscopy to detect CBDs in GUVs formed of POPC/Chol mixtures. CBDs were detected only when the Chol concentration in the mixture was equal to or greater than 75% (Figure 6) [20]. 

DiIC_18_(3) (PL analog) and Bodipy Chol (Chol analog) fluorescent probes were used for the labeling (see Figure 1 for their structures). From our previous studies on MLVs produced by the RSE method, we know that CBDs should appear at a 50 mol% Chol concentration [21]. This indicates significant Chol demixing when producing GUVs using electroformation protocols containing a dry lipid film step during preparation. Interestingly, confocal microscopy images of GUVs revealed very large CBDs [20]. This is in contrast with evaluations suggesting that the CBDs discriminated by EPR are rather small [60]. This discrepancy could possibly be assigned to different systems used for observation, as EPR measurements were performed on MLVs with a curvature much greater than that of GUVs. We assume that in GUVs, smaller individual CBDs coalesce to form a single large CBD. Another unexpected observation is that the CBDs were always detected on the top of GUVs in what is indicated as a lack of a fluorescence signal originating from the DiIC_18_(3) probe that is presented in the middle row of Figure 6, when the Chol/POPC mixing ratio is higher than 3. We proposed that this occurs due to gravitational forces. Because the surface area density of the CBD is ~25% lower than that of the *l*_o_, the gravitation force will orient GUVs with the less dense surface on the top. When we flipped the sample upside down, the GUVs reoriented so that the CBDs could be found on the top again [20].

## 3. SLBs with High Chol Concentrations

### 3.1. SLBs Preparation

#### 3.1.1. Vesicle Fusion SLBs

The advantage of using monolayers is the ability to control the lipid coverage, and a disadvantage is that it only contains one membrane leaflet, so it does not adequately represent the PM. The SLBs contain two lipid leaflets on a solid support, with a 1–2 nm water layer between the surface and the membrane. Different substrates can be used for the fabrication of SLBs, but not all substrates are compatible with all lipid compositions. Substrates that are used the most are silica and glass, but mica, gold, silicon, titanium oxide and others have also been experimented with [61].

**Figure 7:**
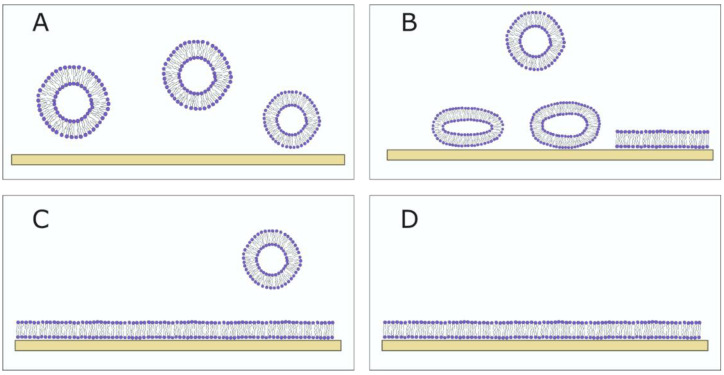
The SLB formation using vesicle fusion method. (**A**) Vesicles float in the bulk solution. (**B**) If the vesicle–substrate interaction is sufficiently strong, the adsorbed vesicles rupture on the substrate. (**C**) The SLB forms, and the unadsorbed vesicles are washed away using a buffer. (**D**) The fully formed SLB.

Different methods can be used for the formation of SLBs. The most widely used method today is vesicle fusion, pioneered by McConnell et al. (Figure 7). The method involves spreading SUVs or LUVs on a hydrophilic surface and their subsequent rupturing in order to form an SLB [62]. It is a fast and easy method but depends on different aspects, such as the vesicle size (a smaller size leads to easier vesicle rupturing), lipid composition (charge of lipids, Chol concentration and saturation degree of the lipids), temperature, the type of substrate and ionic strength [61,63,64,65]. Plasma treatment is most commonly used for surface hydrophilization to promote the rupture of vesicles. Vesicles are adsorbed onto the surface until a critical concentration is achieved, after which the vesicle rupture occurs. Around 1 h is needed for this process to occur [66]. Lipids are then reorganized into a bilayer that is separated from the surface by a thin layer of water. The sample is washed with a buffer to remove unadsorbed vesicles.

It has been shown that using vesicles containing more than 20 mol% Chol makes it harder for the vesicle rupture to occur due to the vesicle heterogeneity and increased rigidity [61]. Some reagents and buffers can be used to alleviate this problem and promote vesicle rupture. NaCl ions and divalent CaCl_2_ cations are often used to promote vesicle rupture [64,67] for SLBs with a Chol content above 20 mol% [67,68,69]. Furthermore, the vesicle lipid composition does not have to be equal to that in the SLB. To quantify the cholesterol fraction in the SLBs, sterol removal by methyl-β-cyclodextrin can be performed and quantified using the Sauerbrey relationship on the quartz crystal microbalance with dissipation monitoring (QCM-d) data [70]. Tabai et al. showed that SLBs had a lower Chol concentration compared to vesicles (20 mol% in vesicles compared to ∼10 mol% in the SLB) [71].

#### 3.1.2. Solvent-Assisted and Bicelle-Mediated SLBs

**Figure 8:**
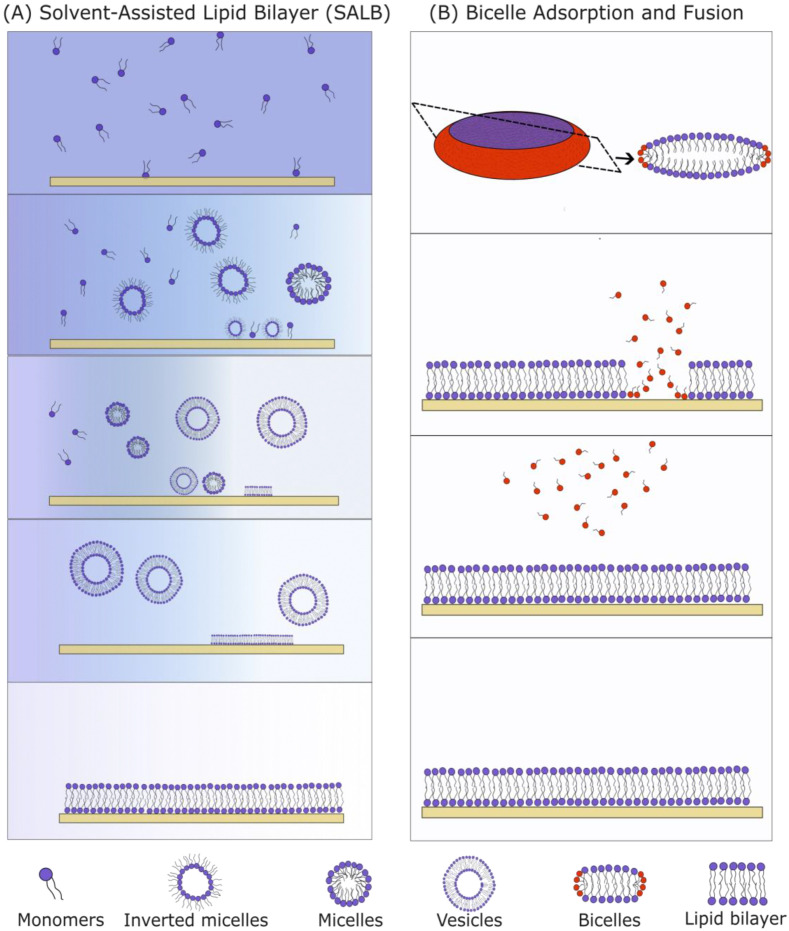
Comparison of the SALB (**A**) and bicelle method (**B**) for formation of SLBs. (**A**) The SALB method begins when long-chain PLs are dissolved in a water-miscible organic solvent. The organic solvent is gradually exchanged with the aqueous solvent. During the solvent exchange, the lipids first assemble into inverted micelles and later into micelles and vesicles. These attach themselves to the surface and rupture to form an SLB. (**B**) Bicelles are disk-like structures containing both long-chain (blue) and short-chain (red) lipids. If there is an attractive force between the substrate and the bicelles, they are adsorbed onto the surface and fuse together. Long-chain PLs remain attached to the substrate, and short-chain PLs are washed away in the form of monomers using a buffer.

Two methods suitable for the formation of SLBs with high Chol content are the solvent-assisted lipid bilayer (SALB, Figure 8A) method and the bicelle-mediated method (Figure 8B). The SALB method incorporates a step principally similar to the one used in the RSE as it involves a gradual exchange of organic and aqueous solutions. Hohner et al. first created a phospholipid SLB using the SALB method by dissolving the PLs in isopropanol and gradually increasing the water content [72]. Depending on the lipid composition, temperature and water content, different lipid structures were formed (micelle, inverted micelle, unilamellar and multilamellar vesicles). During the micelle-to-vesicle transition, the lipid bilayer is formed on a solid substrate. Various protocol parameters (flow, total lipid concentration, lipid content, etc.) need to be regulated for successful SLB production [70]. The solvent exchange velocity needs to be compatible with the velocity of the SLB formation. The total lipid concentration needs to be controlled as well. An overly low amount of lipids leads to incomplete surface coverage (“islands” can be detected), and a surplus of lipids leads to the formation of more than one lipid bilayer. Moreover, just like in vesicle fusion, a surface treatment is recommended for better SLB formation. Using the above approach, a 1,2-dioleoyl-*sn*-glycero-3-phosphocholine/Chol SLB with different Chol concentrations up to ~50 mol% was created successfully [68]. The SLBs were labeled with the rhodamine-modified 1,2-dihexadecanoyl-*sn*-glycero-3-phosphoethanolamine (Rhodamine-DHPE) fluorescent probe which prefers the fluid phase. Using epifluorescence microscopy, dark spots were visible. Considering the fluorescence dye preferences, and the fact that increasing the Chol concentration led to an increase in the area of the dark spots, these spots were hypothesized to be cholesterol-enriched phases. The FRAP experiments showed a near-complete recovery 1 min after the photobleaching, proving that the Chol-depleted phase is laterally fluid. The AFM experiments showed a 1.5 nm difference in height between the phospholipid-rich domain (∼4.5 nm) and the dark spots (∼3 nm). This value is consistent with the values obtained using X-ray diffraction on highly ordered MLVs [54].

Bicelle-mediated SLB formation is another approach that can be used for the fabrication of SLBs with a high Chol content. Bicelles are lipid structures with a disc-like shape, containing a mixture of long-chain and short-chain lipids (Figure 8B). The first publication on bicelle-mediated SLBs was conducted by Zeineldin et al. in 2006, using 1,2-dipalmitoyl-*sn*-glycero-3-phosphocholine (DPPC) and 1,2-dihexanoyl-*sn*-glycero-3-phosphocholine lipids [73]. The main advantage of the method is the ease of the bicelles preparation. A dry lipid film containing a mixture of long-chain and short-chain PLs is hydrated in an aqueous solution and then vortexed. After mixing, the solution is frozen in liquid nitrogen, heated in a water bath at around 60 °C and then vortexed again. Usually, five cycles are needed for the fabrication of bicelles. The obtained suspension is subsequently deposited onto a hydrophilized substrate. Bicelles get adsorbed, they rupture and then a buffer is used to wash away the short-chain lipids and unadsorbed bicelles. The short-chain PLs make bicelles softer compared to vesicles, so they rupture more easily. 

The QCM-d is often used to control the SLB formation. It is a surface-sensitive technique for the determination of the material mass on a piezoelectric crystal quartz sensor. Resonance is achieved when the thickness of the quartz crystal is an odd integer of the half-wavelength of the induced wave, so different overtones (n) can be measured. Changing the frequency gives us information about the mass adsorbed to the crystal. For a successful SLB formation, the change in frequency should be Δf/n = −25 Hz. The energy loss (dissipation) can also be measured using the QCM-d. It is quantified by measuring the oscillation decay time of the quartz crystal after the alternating potential is turned off and depends on the viscoelastic properties of the mass on the crystal. The dissipation for a successful SLB formation should be ΔD/n = 1 × 10^−6^. A combination of Δf and ΔD from measurements of several overtones can be extracted through data modeling. Parameters such as the adsorbed mass, film thickness and viscoelastic properties can be calculated. For a better understanding of the QCM-d, we recommend a detailed review by Allasi et al. [74].

The successfulness of the SLB formation using bicelles depends on the ratio of the long/short-chain PLs (q-ratio), total lipid concentration, lipid composition, type of substrate and NaCl concentration [73,75,76]. Using epifluorescence microscopy, it has been shown that bicelle-mediated SLBs with high Chol content form homogenous SLBs, unlike the SALB method where there are regions with no signal. Although QCM-d results imply the existence of unruptured bicelles on the surface of SLBs containing 40–60 mol% Chol, epifluorescence microscopy could not confirm the same result. On the contrary, regions with unruptured vesicles are seen as bright spots using epifluorescence microscopy. The inability to detect unruptured bicelles is explained by their disk-like shape which allows them to blend in better with the SLB patches.

### 3.2. Experimental Techniques Utilizing SLBs with Particular Focus on CBDs

#### 3.2.1. X-ray Diffraction

**Figure 9:**
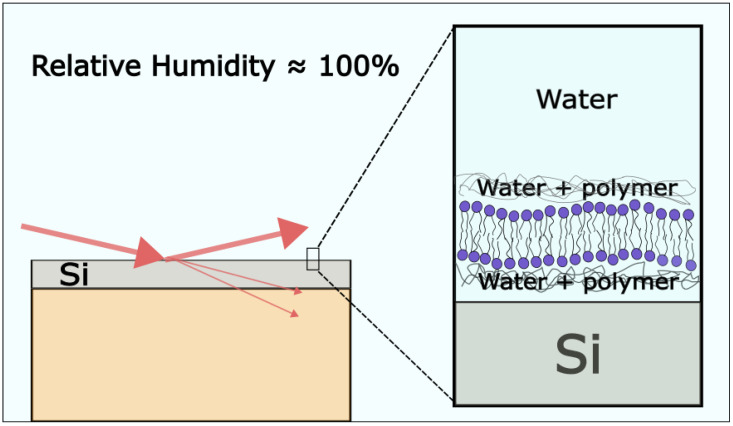
Schematic drawing of grazing-incidence X-ray diffraction measurements of a highly hydrated polymer-cushioned SLB. The figure is based on an image from ref. [78].

Ziblat et al. have examined the formation of CBDs on a polymer-cushioned lipid bilayer using grazing-incidence X-ray diffraction measurements. They introduced a new humidity control method for grazing-incidence X-ray diffraction experiments (Figure 9). In short, using the Langmuir–Blodgett/Langmuir–Schaeffer method, they deposited the bilayer in the chamber and evaporated the sample to 42% relative humidity. The relative humidity was increased by cooling the sample to 6.7 °C, and in these conditions, the relative humidity above the sample was 95.7%. The bilayer was sandwiched with two thin layers of polyethyleneimine. The purpose of the polymer is to keep the bilayer hydrated and allow bilayer components for free diffusion, thus better reproducing biological conditions. A different composition of the lipid bilayer has been investigated with Chol, SM, DPPC or ceramide. The Chol concentration at which they detected CBDs for the SM/Chol mixture was 38 ± 3 mol%; for DPPC/Chol, it was 54 ± 3 mol%; and for Ceramide/Chol, it was 45 ± 5 mol% [77,78,79].

#### 3.2.2. Atomic Force Microscopy

Khadka et al. [67] used AFM to investigate the mechanical properties of POPC/Chol SLBs with Chol concentrations ranging from 0 to 75 mol%. The SLBs were made using the vesicle fusion of SUVs which were prepared using the RSE method. No direct AFM detection of CBDs was made in SLBs with a Chol content higher than 50 mol%. This was probably due to the AFM tip having a larger area than the CBD. The surface roughness of the SLBs was decreasing until a Chol content of 60 mol%, and it started to increase above that level. The increase above 60 mol% is explained by the possible formation of CBDs [67]. Using force spectroscopy, a single puncture event was obtained for SLBs with Chol concentrations up to 50 mol% and for two events above that concentration. The first puncture event represents the coexisting *l*_o_ and CBDs and the second one is thought to represent the “CBD water pocket” formed on the water layer above the surface. Because of the difference in height between the CBD and *l*_o_, an additional buffer thickness, which acts as an additional resistance for the AFM tip, should be present between the CBD and the surface [67].

#### 3.2.3. Fluorescence Microscopy

FRAP is a technique used for the investigation of lateral mobility and the distribution of lipids in an artificial membrane [80]. Litz et al. [81] made SLBs with a high Chol content (30–66 mol%) using vesicle fusion. Most commonly, SUVs or LUVs are used for the vesicle fusion method, but in their experiment, they used GUVs to form the SLBs. The methyl-β-cyclodextrin was used for the removal of Chol from the SLBs. Two populations of Chol with different properties were found, first with low and the second with high surface accessibility. This finding is consistent with the prediction of the CBD model of the cholesterol–phospholipid interaction in the lipid bilayer. The Chol inside the CBDs would be less shielded from the aqueous phase compared to those inside the *l*_o_. The characteristic Chol fraction at which Chol accessibility begins to increase is in correspondence to the CSAT [81].

#### 3.2.4. QCM-D

FRAP and the QCM-d are often-used methods for controlling the formation of SLBs. Sut at al. [82] have successfully formed SLBs using bicelles with mixtures containing up to a 30 mol% Chol concentration. However, when increasing the Chol content further, some bicelles did not rupture, so incomplete SLBs formed with adsorbed unruptured bicelles. The QCM-d data show a successful SLB formation for 10 and 20 mol% of Chol. At 30–60 mol% of Chol, the QCM-d data indicated incomplete SLB formation. Interestingly, the results showed a smaller Δf for 60 mol% compared to 50 mol% with the same ΔD shifts. The authors suggest that this is due to the formation of CBDs which have a different local thickness, causing a change in the Δf without changing the global viscoelasticity of the membrane (determined by ΔD) [83].

## 4. Properties of CBDs

The Gibbs phase rule, f = c − P + 1 (f, degree of freedom, i.e., the largest number of thermodynamic parameters such as temperature and pressure that can be varied simultaneously; c, the number of components; P, the number of phases, assuming that in all phases the membrane is under the same pressure), must be obeyed for all the regions of the phase diagram and in all the indicated phase boundaries. The Gibbs phase rule has imposed a few immediate consequences (limitations) regarding the effects of Chol on the physical properties of the fluid-phase membrane. One of them is that the Chol concentration that can be accommodated within a certain phase has an upper limit. For the *l*_o_ phase, this limit is 50 mol% Chol. The addition of Chol above this concentration leads to the formation of pure CBDs within the *l*_o_ phase. However, the newly formed CBD within the *l*_o_ phase cannot be considered as a new phase because it would break the Gibbs phase rule. The single-phase region (*l*_o_ phase with CBDs) is called a structured [84], or a dispersed [85], *l*_o_ phase. We define the Chol concentration (limit) at which the *l*_o_ phase is saturated with Chol as the CSAT (or at which the CBDs start to form). The total amount of Chol that can be accommodated by the structured *l*_o_ phase (both in the PL bilayer saturated with Chol and in CBDs) is defined as the CST. This limit occurs at 66 mol% of Chol [86]. Above this limit, Chol crystals are formed as a new phase in the Chol/PL mixture. Thus, above this limit, two phases coexist, a structured *l*_o_ phase and Chol crystals [87]. It was shown that in model membranes, the CSAT as well as the CST are decreased by the presence of polyunsaturated chains in the phospholipid bilayer [88]. The peroxidation of phospholipid unsaturated acyl chains has also been linked to a decrease in the CSAT [89,90] and CST [91]. 

Chronologically, about two decades ago, the first reports appeared that the pure bilayers of Chol were detected using X-ray diffraction and supported bilayer membranes formed from distinct mammalian cell types: arterial smooth muscle cells and ocular lens fiber cells [27,92]. According to the authors, these domains were formed by anhydrous Chol molecules, and all the domains possessed a structure as rigid as the bilayers in Chol crystals. Because of that, they named these domains “cholesterol crystalline domains”. Ten years later, the authors of this review detected pure CBDs in membranes oversaturated with Chol by using the saturation recovery EPR method and multilamellar liposomes. The results showed that the rigid cholesterol crystalline domains are not the same as highly fluid CBDs [57]. This highly dynamic structure of the CBD was additionally confirmed by molecular dynamic simulations [88]. Furthermore, both approaches indicated that the Chol –OH groups in CBDs are highly accessible to water molecules; thus, this bilayer cannot be formed by anhydrous Chol molecules. Finally, using fluorescence probe molecules and GUVs, it was unambiguously confirmed that CBDs are transmembrane structures and not separately formed in each bilayer leaflet [20].

We think that the CBD with its unique structure and properties is not yet appreciated enough in membrane research. Additionally, of those properties indicated above, the most significant one is that the presence of the CBD ensures that the surrounding PL bilayer is always saturated with Chol. We can state that the CBD forms the buffering capacity for Chol in the surrounding phospholipid bilayer. Most significantly, the saturation of PL bilayers with Chol ensures that the membrane physical properties of these bilayers (including profiles of the acyl chain order, membrane fluidity, hydrophobicity and oxygen diffusion-concentration product) become consistent and independent of the compositions. It is observed for a membrane made of a single PL, PL mixtures and membranes made of total lipid extracts from intact membranes. The saturation with Chol smooths the bilayer surface. Moreover, the CBD itself forms a significant barrier to oxygen transport [57]. Due to the presence of CBDs in all human fiber cell membranes, the Chol content in these membranes is always high enough to saturate the lens fiber cell membranes regardless of the age of the cell [60]. This ensures the homeostasis of the PMs, fiber cells themselves and entire eye lens [24,25,26]

Using the RSE method for the formation of multilamellar liposomes (Section 2), we were able to show that the formation of CBDs precedes the formation of Chol crystals [21,93]. These results allowed us to extend the phase diagram for Chol/1,2-dimyristoyl-*sn*-glycero-3-phosphocholine mixtures presented by Almeida et al. [87] to greater Chol contents in the bilayer [21]. Moreover, based on these results, a phase diagram was proposed, showing how a mixture of different PLs can affect the CSAT and CST, and it was hypothesized that CBDs, at a further increase in the Chol content in the bilayer, can collapse to form Chol seed crystals which further grow to Chol microcrystals [94]. Below, we will discuss how the effects of these CBD properties and the CBDs themselves affect the surrounding PL bilayer and the functions of the different membranes in the human organism.

## 5. Understanding of CBD Functions in Model Membranes Helps to Understand Its Functions in Biological Membranes

In Section 4, we summarized the properties of CBDs obtained with the different membrane models and techniques described in Section 2 and Section 3. Here, we will only indicate the membrane properties which are significant for understanding the CBD’s function and high (saturating) Chol content in a human organism. To the best of our knowledge, the extremely high Chol content and appearance of CBDs plays a positive role only in the eye lens fiber cell plasma membranes. There, CBDs support the normal physiological function by helping to maintain lens transparency and preventing the development of a cataract [24]. In membranes of other organs and tissues, the appearance of CBDs is treated as a sign of pathology [25,94].

The positive functions of CBDs in fiber cell plasma membranes are already discussed in detail in [24]. Here, we emphasize these functions which were obtained using appropriate membrane models and techniques. We think that the application of SR EPR spin labeling with the use of multilamellar liposomes gave very significant results. It enabled the measurement of physical properties of the PL bilayer surrounding the CBDs (and thus saturated with Chol). (1) The results show that the physical properties of these PL bilayers surrounding the membrane-integral proteins are consistent and independent of the PL composition of the bilayer. These properties ensure the proper functioning of the eye lens fiber cell membranes (ensure their homeostasis) through drastic changes in the PL composition that occurs with age. It is especially significant because human fiber cells undergo minimal cell turnover, so the same membrane-integral proteins are immersed in the lipid bilayer with drastic changes in the PL composition occurring throughout the human life. Thanks to the presence of CBDs and the saturation of this bilayer with Chol, the effects of these drastic changes are minimized, and proteins are always surrounded by a lipid bilayer with consistent physical properties. (2) Another significant function of CBDs is maximizing the membrane hydrophobic barrier. That way, the permeation of polar molecules in and out of fiber cells can be tightly controlled through gap junctions built of connexins Cx43, Cx46 and Cx50 [95] and water channels built of AQP0 [90]. The saturation of the PL bilayer with Chol is necessary to fulfill this condition. These membranes are built mainly by saturated sphingolipids and dihydrosphingolipids, and without Chol, they would display a very low hydrophobic barrier. (3) It is known that any increase in the oxygen partial pressure inside the lens often results in cataract development. One of the mechanisms helping to keep low oxygen partial pressure inside the lens is the formation of barriers to oxygen permeation across the layers of fiber cells. By saturating the PL bilayer with Chol, CBDs significantly increase the barrier to oxygen permeation. Additionally, the CBD itself has very low oxygen permeability. All these factors indicate that high Chol content and CBDs are needed, and even necessary, to protect the lens against oxidative stress (4). Furthermore, the smoothing of the membrane surface by the saturating content of Chol should decrease the light scattering and help to maintain the lens transparency. These conclusions were made from the results obtained with SR EPR spin labeling using multilamellar liposomes. Most of them were confirmed by molecular dynamic simulations of PL bilayers and CBDs properties [88]. 

α-crystallin, the major protein found in the human eye lens cytoplasm, works as a molecular chaperone by preventing the aggregation of proteins and thus maintaining lens transparency [96]. With age, the content of membrane-bound α-crystallin increases at the expense of its decrease in the cytoplasm. These processes are accompanied by increased light scattering which compromises lens transparency. Using continuous wave EPR spin labeling and SUVs as a membrane model, Mainali’s group showed that high Chol content and the presence of CBDs inhibits the binding of α-crystallin to membranes made of the major PLs of the eye lens fiber cell plasma membranes. They showed that the same conclusion is valid for membranes (SUV) made of the total lipids extracted from bovine cortical and nuclear lenses [97]. These results present another mechanism through which high Chol and CBDs protect the eye lens against age-related cataract development. However, it should be noted that the SUVs used in these investigations were made using the RSE method followed by probe-tip sonication, and it was not yet confirmed that CBDs can be formed in SUVs with their high curvature. This is an open problem that is significant for the use of SUVs prepared from lipid mixtures with a high Chol/PL mixing ratio.

The formation of CBDs and Chol crystals in membranes of most organs and tissues is considered a sign of pathology [22,27]. Thanks to the application of the RSE method for the formation of multilamellar liposomes and with the use of SR EPR and differential scanning calorimetry techniques, we were able to show that when the Chol content in the phospholipid bilayer exceeds the CST [24,25,27] Chol crystals form, presumably outside the membrane [21,93]. Additionally, the peroxidation of PLs lowers the amount of Chol needed for CBDs and Chol crystals to start to form [89,90]. These are the conditions driving the development of atherosclerosis (high-Chol level and oxidative stress) in which membranes oversaturated with Chol are no longer able to support some of the CBDs. Consequently, the CBDs detach from the membrane and collapse outside of the membranes in the form of Chol aggregates that can become crystal nuclei and, in time, convert into Chol microcrystals. Chol microcrystals activate inflammasomes, thereby stimulating immune responses and initiating inflammation that may lead to the development of atherosclerosis.

In the eye lens, the presence of CBDs is beneficial, and the appearance of Chol crystals in lenses at old age does not compromise lens transparency [22]. The lens is avascular and, soon after formation, its fiber cells lose their intracellular organelles [98,99]. These factors protect the lens from the harmful induction of inflammasomes by Chol crystals as well as from the initiation of the inflammatory cascade. Thus, inflammation does not appear to be involved in cataract formation in the eye lens.

## 6. Conclusions and Perspectives

We presented the protocols for the preparation of membrane models at Chol concentrations above the CSAT and discussed the problems that may be encountered. GUVs, MLVs and SLBs have been used in CBD studies. The most commonly used method for GUVs production is electroformation. The traditional electroformation protocol contains a step where the lipid film is in a dry state. This induces Chol precipitation in the form of crystals—an issue known as the Chol demixing artifact. These crystals are not incorporated into the GUV bilayer, resulting in a decreased Chol concentration compared to the initial mixture. The dry phase can be bypassed using the RSE method. However, the resulting solution contains only MLVs and not GUVs. Baykal-Caglar et al. have succeeded in reconciling these two approaches to produce GUVs using electroformation without the dry lipid film step. They achieved this using the RSE solution to produce damp lipid films which were used in electroformation.

SLB formation at high Chol contents is also challenging. Vesicle fusion is the most commonly used method but can be problematic at Chol concentrations above 20 mol%. Recent studies suggest that specific ions and buffers can be used to promote a vesicle rupture, potentially solving this issue. The RSE method can be very useful for the production of SLBs, especially as a part of the vesicle fusion protocol. As previously stated, this method of preparation is required to form vesicles with high Chol content. The SALB and bicelle methods can be used as alternatives for SLB formation with Chol contents near and above the CSAT. Using fluorescence microscopy, dark and bright spots could be seen in SLBs prepared by the SALB method, whereas no dark spots could be detected when the bicelle method was used. 

CBDs were detected using different techniques applied to different model systems. They were directly detected using confocal microscopy of electroformed GUVs, EPR studies on MLVs and intact membranes and X-ray measurements of polymer-cushioned SLBs and highly oriented MLVs. Measurements indirectly implying CBD formation have also been obtained using AFM and QCM-d on SLBs.

Using confocal microscopy on GUVs with a Chol concentration above the CSAT, large CBDs were observed. This is in contrast with much smaller CBDs discriminated by the EPR. It is suggested that the difference appears due to EPR studies being performed on MLVs which have a much higher curvature compared to GUVs. EPR studies provide information about the lateral organization of the lipid bilayer. The order of the PL acyl chains, fluidity and hydrophobicity of the bilayer were obtained as a function of the bilayer depth on the model, the lens lipid and intact membranes. At Chol concentrations higher than the CSAT, two domains were observed within the bilayer, representing the *l*_o_ phase and CBD, respectively. X-ray diffraction methods were also used to measure the properties of pure Chol domains but were unable to explain the Chol molecular dynamics within the bilayer. These experiments have been performed on highly oriented MLVs and polymer-cushioned SLBs.

Because CBDs appear at very high Chol concentrations (above ~50 mol% in PLs), the existing techniques for the preparation of model membranes are usually not equipped to handle such conditions. Although CBDs were already detected in GUVs, that study used traditional electroformation, resulting in the Chol demixing artifact. Consequently, CBDs could not be detected below a Chol concentration of 75 mol% in the initial mixture. Although not tested on Chol concentrations above the CSAT, modifications of the electroformation protocol by Baykal-Caglar et al. should enable the production of GUVs with well-controlled Chol contents. This should allow for a comparison of CSATs with different membrane models (for example, MLVs). The bicelle and SALB methods look the most promising for the production of SLBs with a high Chol concentration. These new methods can be utilized to study CBD properties in order to further elucidate its role in the membrane.

Regarding the biological function of CBDs in PMs, the presence of CBDs in the eye lens membrane has been shown to be beneficial. *l*_o_ surrounded by CBDs have consistent physical properties regardless of the PL composition. This is important for the homeostasis of the eye lens, whose PL composition changes with aging. The proteins in the fiber cells of the eye lens are always surrounded by the PLs with constant physical properties. Another beneficial property of CBDs is the maximization of the hydrophobic barrier of the membrane, so that the movement of polar molecules within the fiber cells is regulated by gap junctions and water channels. Increased oxygen partial pressure also promotes cataract formation, and CBDs saturate *l*_o_ to keep the oxygen barrier high during aging. The smoothing of the membrane surface by saturation with Chol reduces light scattering. In other membranes and tissues, CBDs and Chol crystals are indicative of a pathological condition.

## Data Availability

Not applicable.

## Abbreviations

AFM	Atomic force microscopy
ASL	Androstane spin label
CBD	Cholesterol bilayer domain
Chol	Cholesterol
CSL	Cholestane spin label
CST	Cholesterol solubility threshold
CSAT	Cholesterol saturation threshold
DPPC	1,2-dipalmitoyl-*sn*-glycero-3-phosphocholine
DiIC_18_(3)	1,1′-dioctadecyl-3,3,3′,3′-tetramethylindocarbocyanine
FRAP	Fluorescence recovery after photobleaching
GUV	Giant unilamellar vesicle
*l*_d_	Liquid-disordered domain
*l*_o_	Liquid-ordered domain
LUV	Large unilamellar vesicle
MLV	Multilamellar vesicle
MVV	Multivesicular vesicle
OLV	Oligolamellar vesicle
PL	Phospholipid
PM	Plasma membrane
POPC	1-palmitoyl-2-oleoyl-glycero-3-phosphocholine
QCM-d	Quartz crystal microbalance with dissipation monitoring
RSE	Rapid solvent exchange
SALB	Solvent-assisted lipid bilayer
SM	Sphingomyelin
SLB	Supported lipid bilayer
SR EPR	Saturation recovery electron paramagnetic resonance
SUV	Small unilamellar vesicle
